# Supplementary material for: Assessment of patient safety culture and associated factors among healthcare professionals in public hospitals of Bahir Dar City, Northwest Ethiopia: A mixed-methods study
Source: PLoS One. 2024 Nov 7;19(11):e0313321. doi: 10.1371/journal.pone.0313321 (PMC11542836; doi:10.1371/journal.pone.0313321)
Supplement: Attachment — Submitted filename: Response to Reviewers.docx [file pone.0313321.s001.docx]

**“Response to reviewers”**

We are grateful to the reviewer for taking time to carefully review the manuscript and give detailed and constructive comments, which has greatly helped to improve this paper. Below is our point-by-point response to each respective comment.

| **Reviewers** | **Comments** | **Responses** |
| --- | --- | --- |
| **Reviewer#1** | General editing for language to make it easier for reader | We appreciate the reviewer for taking time to carefully review the manuscript. Accordingly, throughout the manuscript, we have revised and edited about grammar, spelling, punctuation, consistency and clarity |
|  | Add limitation of the study | We agree with the reviewer ‘invaluable comment. Accordingly, we added the limitation of the study |
| **Reviewer#2** | Graphs/tables about composite score | Thanks for the kind comment. We addressed the comment accordingly |
|  | Information about the HSOPS results | Thank you for your constructive comment. We addressed the comment accordingly |
|  | Survey administration Language | Thank you for your constructive comment.  Since the data collected from healthcare professionals, survey was administrated in English version, which is more convenient than local language. |
|  | Was the current, or a previous, SOPS translation guide followed? | Thank you for your kind comment.  **Yes** , we have had followed Translation Guidelines for the AHRQ Surveys on Patient Safety Culture™ (SOPS®) |
|  | Which version of HSOPS was used? | Thank you for your constructive comment.  We have used HSOPS tool Version 1.0 |
|  | Did you get permission to use the survey internationally | Thank you for your positive comment.  **Yes**, we have gotten permission from Agency for Healthcare Research and Quality (AHRQ) by Mr. Howard Holland, Director, and AHRQ’s Office of Communications. |
|  | Stepwise regression analysis | Thank you for your constructive comment. But,  Even though selection using statistical significance(p value<0.20) for stepwise regression analysis is problematic, including all results in regression analyses did not bring any difference on the outcome in this study |
|  | De-identification | Thank you for invaluable and positive comment.  We addressed the comment accordingly and rewrite it in a better personal identification form |
|  | About p-values | Thank you for valuable comment.  We addressed the comment accordingly |
|  | Data sharing | Thank you for your positive comment.  We addressed the comment accordingly |
| **Reviewer#3** | Grammar, spelling mistake sand consistency | Thank you for your constructive comment. Accordingly, throughout the manuscript, we have revised and edited it about grammar, spelling, punctuation, consistency, clarity, and relevancy |
|  | WHO reference with ideas | Thank you for your constructive comment. We addressed the comment accordingly and replaced the paragraph with other paragraph which explained the idea in a sound manner |
|  | Measurement/score | We agree with the reviewer’s comment. Accordingly, we addressed the comment and incorporate how to measure and interpret HSOPS |
|  | Information relevance for the topic | Thank you for your constructive comment. We addressed the comment accordingly |
|  | Clarity issue | Thank you for your constructive comment. We have tried to address the comment accordingly |
|  | Sampling techniques | Thank you for comment. Stratified sampling technique was used to select the 420 study participants among 3 public hospitals. By using proportional allocation formula, simple random sampling technique was applied to select participants from each stratum |
|  | Why not all healthcare professionals included? | Thank you for your constructive comment. Almost all healthcare professionals included in this study except public health officer and physiotherapy which is very small in number |
|  | Participant experience difference between qualitative and quantitative | Thank you for your valuable comment. We have agreed that **in case of quantitative study** healthcare professionals who had worked at least for 6 months(considered as permanent employee), should be included where as i**n case of qualitative study,** in order to get detail idea/information/explanation, purposively we agreed that work experience should be at least 5 years |
|  | How did the participants get access to the questionnaire | Thank you for your constructive comment.  Data were collected through self-administered  (paper-based) data collection techniques, and three Bachelor of Science-graduated nurse collected the data |
|  | If thematic Analysis conducted, How many codes where created? What are the names of the codes? | Thank you for your constructive comment  By using the open code software version 4.02, we have created about 18 codes, and finally theme it in to three main category based on the source of the problem(organizational related , healthcare professional related , and patient related) |
|  | Methodology for qualitative study | Thank you for your invaluable comment.  Specifically, we used **Ethnographic approach** for qualitative study.  In general, we have used convergent parallel mixed method; we collected quantitative and qualitative data at the same time, and then analyze them separately. After both analyses are complete, we have tried to compare our results to draw overall conclusions |
|  | What is the need of supportive letter | Thank you for your constructive comment .In order to collect the data, supportive letter to specific hospital is mandatory beside ethical approval. |
|  | Streamline the results section | Thank you for your positive comment. We addressed the comment accordingly |
|  | Focus on triangulation | Thank you for your valuable comment. We addressed the comment accordingly |
|  | Unclear statements | Thank you for your constructive comment. We addressed the comment accordingly |
|  | Assumptions reference | Thank you for your constructive comment. We addressed the comment accordingly |
|  | What is called department medicine? | Thank you for your constructive comment. It is corrected as internal medicine |
|  | Table1. the use of upper and lower case letters inconsistent | Thank you for your constructive comment. We addressed the comment accordingly |
|  | Safety box, essential medicine | Thank you for your kind comment. Safety box for needle and sharp material  Essential medicine means medicine those used for emergency situation in hospital and frequently used |
|  | Figure 1: mix up with figure 3 | Thank you for this suggestion. We addressed the comment accordingly |
| **Reviwer#4** | Difference between patent safety and patient safety culture | Thank you for your constructive comment. We addressed the comment accordingly |
|  | Triangulation is not explained in the paper | Thank you for your constructive comment. We addressed the comment accordingly |
|  | First sentence is not clear to me | Thank you for your constructive comment. We addressed the comment accordingly. |
|  | What does the positive response rate | Thank you for your constructive comment. We addressed it by adding measurement |
|  | What does the prevalence in the 3rd paragraph (57.6%) refer to? | Thank you for your constructive comment. We addressed and corrected it |
|  | What is the aim of the study? | Thank you for your constructive comment.  The aim study is to assess patient safety culture and influencing factors among healthcare professionals in Bahir Dar city public hospitals, Northwest Ethiopia. |
|  | Which qualitative method was used? | Thank you for your invaluable comment.  **Specifically**, we used Ethnographic approach for qualitative study.  **In general**, we have used convergent parallel mixed method; we collected quantitative and qualitative data at the same time, and then analyze them separately. After both analyses are complete, we have tried to compare our results to draw overall conclusions. |
|  | Inclusion criteria for quantitative study | Thank you for your positive comment.  All healthcare professionals who had worked at least for 6 months prior to data collection |
|  | How large was the population of the quantitative survey? | Thank you for your constructive comment. The source population of three public hospitals were 1805 healthcare professionals |
|  | What is the sample representative | Thank you for your constructive comment. It corrected as study participants |
|  | Why experience difference between qualitative and quantitative study? | Thank you for your valuable comment.  We have agreed that in case of quantitative study, healthcare professionals who had worked at least 6 months(considered as permanent employee), should be included where as in case of qualitative study, in order to get detail idea/information/explanation, purposively we agreed that work experience should be at least 5 years |
|  | Why was the survey adapted? | Thank you for pointing this out.  The HSOPS tool was adopted from the Agency for Healthcare Research and Quality in the United States (AHRQ) |
|  | How was the survey conducted | Thank you for your constructive comment.  Data were collected through self-administered  (paper-based) data collection techniques, and three Bachelor of Science-graduated nurse collected the data |
|  | Is Amharic the local language? | Yes, Amharic is the local language |
|  | Were the qualitative interviews conducted by only one person? | No, it was collected by two data collector including principal investigator |
|  | How many researchers worked with the data in total? | Thank you for your constructive comment. A total of 6 researchers are involved in this research including data collectors |
|  | Why were the transcripts translated into English? | Since Amharic is the local language, and in order to communicate with international community, we planned to write and translated into English |
|  | Why was the questionnaire tested in Gondar Hospital? | Thank you for pointing this out.  To determine the feasibility of our research design, and it help us to assess our approach and practice the necessary techniques required for our project |
|  | What is meant by the "supportive letter? | Thank you for your constructive comment  Supportive letter is a letter which is written by an academic institution to specific institution/hospital in order to get permission for data collection beside ethical approval |
|  | What was the dependent variable? | Thank you for your constructive comment  The dependent variable is “***Patient safety culture”*** |
|  | The description of the qualitative results lacks a common thread | Thank you for your constructive comment. We addressed the comment accordingly |
|  | Category of influencing factors | Although influencing factors overlapping each other, we classified them into three major categories (organizational, healthcare professional , and patient related factors) based on their majority contributing (source)factors |
|  | Where are the references for the hypotheses put forward? | Thank you for your constructive comment. We addressed the comment accordingly |
|  | Aim of the study and also the results | Thank you for your constructive comment.  The aim study is to assess patient safety culture and influencing factors among healthcare professionals in Bahir Dar city public hospitals, Northwest Ethiopia |
|  | The tables should be more informative | Thank you for your constructive comment. We addressed the comment accordingly |
|  | The indication of the standard deviation is unusual | Thank you for your constructive comment. We addressed the comment accordingly, and rewrite and corrected it |
| **General Comment#** | Please ensure that your manuscript meets PLOS ONE's style requirement | Thank you for your constructive comment. We addressed the comment accordingly |
|  | Follow online submission form | Thank you for your constructive comment. We addressed the comment accordingly |
|  | Include a caption for figure 1 | Thank you for this suggestion. We addressed the comment accordingly |
|  | Please upload a copy of Figure 3 | Thank you for your valuable comment. We addressed the comment accordingly , and already uploaded |
|  | Please include your tables as part of your main manuscript | Thank you for your constructive comment. We addressed the comment accordingly |
|  | Discussion section could be strengthened | Thank you for your constructive comment. We addressed the comment accordingly |
|  | De-identification of interview quotes could be improved | Thank you for your constructive comment. We addressed the comment accordingly |
|  | Sampling method and representativeness criteria | Thank you for comment. Stratified sampling technique was used to select the 420 study participants among 3 public hospitals. By using proportional allocation formula, simple random sampling technique was applied to select participants from each stratum |
|  | Focus on data triangulation and proper referencing | Thank you for your constructive comment. We addressed the comment accordingly |
|  | Define "safety box." | Thank you for your constructive comment. Safety box for needle and sharp material |
|  | Rationale for different experience requirements in quantitative vs qualitative | Thank you for your valuable comment.  We have agreed that in case of quantitative study, healthcare professionals who had worked at least 6 months(considered as permanent employee) should be included where as in case of qualitative study, in order to get detail idea/information/explanation, purposively we agreed that work experience should be at least 5 years |
