# Supplementary material for: Assessment of patient safety culture and associated factors among healthcare professionals in public hospitals of Bahir Dar City, Northwest Ethiopia: A mixed-methods study
Source: PLoS One. 2024 Nov 7;19(11):e0313321. doi: 10.1371/journal.pone.0313321 (PMC11542836; doi:10.1371/journal.pone.0313321)
Supplement: Attachment — Submitted filename: Response to Reviewers.docx [file pone.0313321.s002.docx]

**Response to reviewers**

We are grateful to the reviewers for their time and effort in carefully reviewing the manuscript and providing detailed and constructive feedback, which has greatly improved the quality of our paper. Below is our point-by-point response to each comment.

| **Reviewers** | **Comments** | **Responses** |
| --- | --- | --- |
| **Reviewer#3** | Please check for continuous format style | We appreciate the reviewer’s careful review. We have revised and edited the manuscript for grammar, spelling, punctuation, consistency, and clarity. |
|  | Proofreading needed in the Abstract | We have revised the abstract to include the major results of this study, which are highlighted in the revised manuscript. |
|  | “Globally, patient harm attributed to unsafe healthcare delivery..." is not clear | We agree with the reviewer’s invaluable comment and have clarified this statement accordingly. |
|  | Avoid duplication of the same paragraphs in the Abstract and Introduction | Thank you for the comment. We have addressed this by removing redundant content to ensure the Abstract and Introduction provides distinct and complementary information. We believe this enhances the clarity and focus of the manuscript |
|  | “Inclusion criteria” – the part "for quantitative study" is not clear | Thank you for your constructive feedback. We have clarified this section in the revised manuscript. |
|  | Clarify “self-administered data collection techniques” | Thank you for your comment. We have clarified this as "participants filled out the questioners independently". |
|  | Clarify language mistakes | We appreciate the reviewer’s attention to detail. The manuscript has been revised for grammar, spelling, punctuation, consistency, and clarity. |
|  | Make clear the description of the section “measurement” | We agree that the original explanation was unclear. We have revised this section to address the comment. |
|  | Make clear "in-depth interview transcription and translation" | Thank you for your valuable comment. We have rewritten this section to improve clarity. |
|  | Why do you have more than two-thirds of male respondents? | We acknowledge the reviewer's concern. However, the higher proportion of male respondents reflects the demographic composition in Ethiopian healthcare institutions, as seen in previous studies in Jimma, Gondar, and Bale. |
|  | Make clear “Data Management Analysis” and “Data quality management | We appreciate the reviewer’s feedback. We have rewritten the “Data processing and analyses” section to include valuable information and clarify these aspects. |
|  | Make monthly salary readable on an international level, like in dollars | Thank you for the suggestion. We have converted the salary figures to US dollars for better readability. |
|  | What is the patient safety grade? | We have incorporated discretion of “patient safety grade” in both the Introduction and Discussion sections to address this comment. |
|  | Qualitative findings: why are all participants not mentioned? | We agree with the reviewer’s comment. We have addressed this by incorporating the relevant details in a table. |
|  | Inconsistent use of "healthcare professional" vs. "health care professional | Thank you for pointing out this inconsistency. We have standardized the terminology throughout the manuscript. |
|  | The result section of the qualitative findings is not well-structured | Thank you for your suggestion. We have revised the Results section, incorporating major comments as highlighted in the revised manuscript. |
|  | Please focus more on triangulating your quantitative and qualitative data | Thank you for your constructive comment. We have rewritten the Discussion section to better triangulate the quantitative and qualitative data. |
|  | Why might the study not be compared to private hospitals? | Thank you for your valuable comment. We have addressed the rationale for not comparing this study with private hospitals in the manuscript. |
| **Reviwer#4** | **Data availability issues:**  1.The values behind the means, standard deviations and other measures reported  2.The values used to build graphs  3. The points extracted from images for analysis. | Thank you for your insightful feedback regarding data availability. In response to the request for the underlying data, we have made the following updates:   - We have uploaded the raw data used to calculate the means, standard deviations, and other reported measures. - The data used to generate all figures and graphs in the manuscript has been made available in the repository. - The points and datasets derived from image analyses have also been included in the supplementary data.   All of the requested data has been uploaded to a public repository, which can be accessed at the following link: : <https://doi.org/10.5281/zenodo.13759397> |
|  | General editing for language and punctuation. | Thank you for your constructive comment. We have revised and edited the manuscript for grammar, spelling, punctuation, consistency, clarity, and relevance. |
|  | Avoid duplication of the same conclusion in the Abstract and at the end. | Thank you for the comment. We have addressed this by ensuring there is no duplication. |
|  | Introduction: the global burden of disease ranking fourteen is unclear? | We agree with the reviewer’s comment. We have clarified this statement in the revised manuscript. |
|  | What is the logic behind the enumeration of the level of patient safety culture? | Thank you for your suggestion. We have revised the manuscript to clarify this. We hope this addresses your concerns and improves the overall clarity and reliability of our work. |
|  | What is meant by "saturation theory"? | We acknowledge the reviewer's concern. We have included a more detailed description of "saturation theory" in the "Sample Size Determination" section. |
|  | What is the role of the nurses who "collected" the data? | Thank you for your valuable comment. We clarified that the data collectors distributed, guided, and collected the questionnaires, ensuring they were completed correctly while maintaining confidentiality. Two supervisors oversaw the data collection process. |
|  | The "ethnographic approach" still seems too broad. | Thank you for your kind comment. We have clarified this as “Studying people and their cultures by observing them in their natural environment…” |
|  | How were interviews recorded and transcribed? Is just one? | We agree that the original explanation was unclear. We have revised the section to specify that "tape-recorded in-depth interviews were transcribed and then translated into English…." |
|  | What is the logic in the enumeration of "Educational Status" and "Profession"? | Thank you for your suggestion. We have clarified this enumeration in the revised manuscript. |
|  | Why is marital status relevant? | Thank you for your constructive comment. Marital status was included because existing literature indicates it can influence work-life balance and, consequently, professional performance. |
|  | What is meant by "... areas with the most potential for improvement"? | Thank you for your insightful feedback. We have revised the measurement and "Data Processing and Analyses" sections to clearly state and operationalize the level of patient safety. |
|  | Avoid word-for-word repetition in the Results and Discussion sections | Thank you for your valuable comment. We have thoroughly reviewed both sections and corrected any redundancies. |
|  | Cite the reference that supports your hypotheses | We agree with the reviewer’s comment. We have added the relevant references to support our hypotheses. |
|  | Conclusion is very weak and repetitive | Thank you for your constructive comment. We have revised the Conclusion section to include more substantial and relevant information. |
| **General Comment#** | Is the manuscript technically sound? | We appreciate the reviewer's careful assessment. We acknowledge the oversight in the original manuscript, regarding the technical sound of manuscript. We believe these revisions have strengthened the technical soundness of the manuscript. We hope this addresses your concerns and improves the overall clarity and reliability of our work. |
|  | **Data availability issues:**  1.The values behind the means, standard deviations and other measures reported  2.The values used to build graphs  3. The points extracted from images for analysis. | Thank you for your insightful feedback regarding data availability. In response to the request for the underlying data, we have made the following updates:   - We have uploaded the raw data used to calculate the means, standard deviations, and other reported measures. - The data used to generate all figures and graphs in the manuscript has been made available in the repository. - The points and datasets derived from image analyses have also been included in the supplementary data.   All of the requested data has been uploaded to a public repository, which can be accessed at the following link: : <https://doi.org/10.5281/zenodo.13759397> |
|  | Is the manuscript presented in an intelligible fashion and written in standard English? | Thank you for your feedback regarding the clarity and language of my manuscript. We appreciate your insights and have made the following revisions to enhance the intelligibility of the text: Grammar and syntax corrections, terminology consistency, simplification of complex sentences, and overall language and style improvement. We believe these revisions have significantly improved the manuscript's clarity. |
|  | Include dual publication, research ethics, or publication ethics concerns | Thank you for raising this important point. We have carefully considered your concerns regarding dual publication, research ethics, and publication ethics. To address these issues, we have ensured the following:   - No Dual Publication**:** The research presented in this manuscript is original and has not been published or submitted elsewhere - Adherence to Research Ethics**:** All research procedures were conducted in accordance with established ethical guidelines, including Institutional Review Board (IRB) approval. - Compliance with Publication Ethics**:** We have followed the guidelines and policies of the journal regarding authorship, plagiarism, and data integrity. We have ensured that all authors have made significant contributions to the research and have been appropriately credited. |
|  | Please include your tables as part of your main manuscript and remove the individual files. | Thank you for your suggestion regarding the presentation of tables. In response to your request, we have now incorporated all tables directly into the main manuscript and removed the individual files previously submitted. |
