# Supplementary material for: Assessment of patient safety culture and associated factors among healthcare professionals in public hospitals of Bahir Dar City, Northwest Ethiopia: A mixed-methods study
Source: PLoS One. 2024 Nov 7;19(11):e0313321. doi: 10.1371/journal.pone.0313321 (PMC11542836; doi:10.1371/journal.pone.0313321)
Supplement: Attachment — Submitted filename: Response to Reviewers.docx [file pone.0313321.s003.docx]

**Response to reviewers**

We are grateful to the reviewers for their time and effort in carefully reviewing the manuscript and providing detailed and constructive feedback. Their insights prompted us to clarify our methodology, strengthen our arguments, and enhance the overall coherence of the paper. We believe these revisions significantly improved the quality of our paper. Below is our point-by-point response to each comment.

| **Reviewers** | **Comments** | **Responses** |
| --- | --- | --- |
| **Journal Requirements** | "Please review your reference list to ensure that it is complete and correct. If you have cited papers that have been retracted, please include the rationale for doing so in the manuscript text, or remove these references and replace them with relevant current references. Any changes to the reference list should be mentioned in the rebuttal letter that accompanies your revised manuscript. If you need to cite a retracted article, indicate the article’s retracted status in the References list and also include a citation and full reference for the retraction notice." | Thank you for your valuable comments and for bringing this important matter to our attention. We have conducted a comprehensive review of all the references cited in our manuscript to ensure that **none are retracted**. Below is a detailed summary of the steps we took to verify the status of each reference:   1. **Article-Level Checks**: We manually reviewed each referenced article to check for any retraction notices. We specifically examined the title, abstract, and full text for footnotes or disclaimers indicating retraction or withdrawal. We also checked if the publisher had updated the article’s title to reflect retracted status (e.g., including “[Retracted]” or “[Withdrawn]”). 2. **Retraction Watch Database**: We used the Retraction Watch database, which maintains an extensive collection of retracted papers, to search for each reference. We searched by article title, author, and DOI on the [Retraction Watch website](http://retractiondatabase.org). No references from our manuscript were flagged as retracted. 3. **CrossRef Retraction Status**: We utilized CrossRef’s retraction search tool to check the retraction status of each reference. By using the DOI of each article, we confirmed that none of the references in our manuscript had an associated retraction notice. We conducted this search via [crossref.org](https://www.crossref.org). 4. **Google Scholar**: As part of our review, we searched for each article on Google Scholar. Google Scholar typically marks retracted articles with “[RETRACTED]” next to the title. No such notices appeared for any of our citations, further confirming that none have been retracted. 5. **PubMed**: Finally, we reviewed the PubMed database, which flags retracted biomedical articles and provides links to retraction notices. We searched for each reference in PubMed and found no indication that any of the articles had been retracted.   **Conclusion**: Based on this thorough review, we confirm that none of the references cited in our manuscript have been **retracted**. As a result, no changes to the reference list are required. We appreciate your careful consideration of this matter and remain committed to ensuring the highest standards of academic integrity. |
| **Editor’s Comments** | Do highlight the changes or revisions done in the revised manuscript and ensure a point-by-point response to the reviewers for further assessment. Do not put track changes or strikethrough manuscript. | We sincerely thank the editor and reviewers for their valuable comments and suggestions.   - We have made all the necessary revisions in the manuscript and highlighted the changes using **bold text.** Track changes or strikethroughs have not been used, in accordance with the editor’s request. Please refer to the revised manuscript for the highlighted sections. - "Please consider rephrasing 'there is a need for mixed-method studies' to make it more assertive in abstract.” - Beyond addressing the reviewers' specific comments, we also made several general revisions to improve the overall quality of the manuscript without track changes or strikethroughs: - We have conducted a meticulous review of the entire manuscript and corrected all punctuation, spacing, and grammatical errors. This includes fixing misplaced commas, ensuring proper spacing after punctuation, and resolving sentence structure issues. - Conducted another thorough proofreading to catch any minor errors that may have been overlooked initially. - Ensured that the entire manuscript adheres to the journal's formatting and stylistic requirements. |
